# Insulin-stimulated phosphorylation of protein phosphatase 1 regulatory subunit 12B revealed by HPLC-ESI-MS/MS

**DOI:** 10.1186/1477-5956-10-52

**Published:** 2012-09-01

**Authors:** Kimberly Pham, Paul Langlais, Xiangmin Zhang, Alex Chao, Morgan Zingsheim, Zhengping Yi

**Affiliations:** 1 Center for Metabolic and Vascular Biology, Arizona State University, Tempe, AZ, USA; 2 Department of Pharmaceutical Sciences, Eugene Applebaum College of Pharmacy/Health Sciences, Wayne State University, 259 Mack Ave., Detroit, MI, USA

**Keywords:** PPP1R12B, Phosphorylation, HPLC-ESI-MS/MS, Insulin signaling, Label-free, Quantification

## Abstract

**Background:**

Protein phosphatase 1 (PP1) is one of the major phosphatases responsible for protein dephosphorylation in eukaryotes. Protein phosphatase 1 regulatory subunit 12B (PPP1R12B), one of the regulatory subunits of PP1, can bind to PP1cδ, one of the catalytic subunits of PP1, and modulate the specificity and activity of PP1cδ against its substrates. Phosphorylation of PPP1R12B on threonine 646 by Rho kinase inhibits the activity of the PP1c-PPP1R12B complex. However, it is not currently known whether PPP1R12B phosphorylation at threonine 646 and other sites is regulated by insulin. We set out to identify phosphorylation sites in PPP1R12B and to quantify the effect of insulin on PPP1R12B phosphorylation by using high-performance liquid chromatography-electrospray ionization-tandem mass spectrometry.

**Results:**

14 PPP1R12B phosphorylation sites were identified, 7 of which were previously unreported. Potential kinases were predicted for these sites. Furthermore, relative quantification of PPP1R12B phosphorylation sites for basal and insulin-treated samples was obtained by using peak area-based label-free mass spectrometry of fragment ions. The results indicate that insulin stimulates the phosphorylation of PPP1R12B significantly at serine 29 (3.02 ± 0.94 fold), serine 504 (11.67 ± 3.33 fold), and serine 645/threonine 646 (2.34 ± 0.58 fold).

**Conclusion:**

PPP1R12B was identified as a phosphatase subunit that undergoes insulin-stimulated phosphorylation, suggesting that PPP1R12B might play a role in insulin signaling. This study also identified novel targets for future investigation of the regulation of PPP1R12B not only in insulin signaling in cell models, animal models, and in humans, but also in other signaling pathways.

## Introduction

Protein phosphatase 1 (PP1) is one of the most abundant serine/threonine phosphatases; it is responsible for most protein dephosphorylation [1-3], which regulates diverse biological processes in eukaryotes. Interactions between catalytic subunits of PP1 (PP1c) and the regulatory subunits of PP1 (PP1R) lead to the formation of numerous PP1 complexes that have unique substrate specificities, distinct subcellular localizations, and various regulatory mechanisms [1-3].

Protein phosphatase 1 regulatory subunit 12B (PPP1R12B), also known as myosin phosphatase target subunit 2 (MYPT2), is one of the regulatory subunits of PP1 and is predominantly expressed in cardiac/skeletal muscle and brain [4,5]. PPP1R12B regulates muscle contraction, cardiac torsion, and sarcomere organization as well as other cellular processes [5]. PPP1R12B contains an RVxF binding motif (residues 53–56), several ankyrin repeats, and a C-terminal leucine zipper domain, all of which are involved in protein-protein interactions [4-7]. In addition, PPP1R12B has 108 serine, 63 threonine, and 16 tyrosine residues, 26 of which have been reported as phosphorylated in the four large phosphorylation databases (http://www.phosphosite.org, phospho.elm.eu.org, http://www.uniprot.org, and http://www.phosida.com). However, only phosphorylation at threonine 646 (Thr646) has been shown to regulate PPP1R12B function [5]. Thr646 was phosphorylated by Rho-kinase in kidney COS7 cells, reducing the activity of the PPP1R12B-PP1cδ complex [5]. Whether Thr646 phosphorylation plays the same inhibitory role in PPP1R12B-PP1cδ complex activity in other cells remains to be determined.

Insulin is a potent anabolic hormone that modulates a wide variety of biological processes. Protein phosphorylation plays a critical role in relaying the insulin signal from initiation at the insulin receptor to the transport of GLUT4 to the plasma membrane. Dysregulated protein phosphorylation events in insulin signaling may contribute to various diseases, such as type 2 diabetes and cardiovascular diseases. Extensive research has been carried out to study the role of kinases in insulin action. However, a mechanism for serine/threonine phosphatase action in insulin signal transduction is largely unknown. In an effort to discover phosphatases that may be involved in insulin signaling, we identified protein phosphatase 1 regulatory subunit 12A (PPP1R12A) as a novel endogenous, insulin stimulated interaction partner of insulin receptor substrate-1 (IRS-1), a well recognized player in insulin signaling, implying that PPP1R12A might play a role in IRS-1 dephosphorylation and insulin signaling [8]. PPP1R12A is an isoform of PPP1R12B with high expression in smooth muscle cells [9]. As mentioned previously, PPP1R12B is predominantly expressed in cardiac/skeletal muscle and brain. Thus, it is possible that PPP1R12B could anchor the catalytic subunit of PP1, PP1cδ, to dephosphorylate IRS-1 in cardiac/skeletal muscle and brain. More recently, we provided a relative global picture of PPP1R12A phosphorylation in CHO/IR cells, and reported that insulin stimulated or suppressed PPP1R12A phosphorylation at multiple sites [10]. It is currently not known whether insulin plays a regulatory role in PPP1R12B phosphorylation. Therefore, in the present study, we used multi-segment high performance liquid chromatography-electrospray ionization-tandem mass spectrometry (HPLC-ESI-MS/MS) to identify and quantify PPP1R12B phosphorylation sites that are regulated by insulin. We utilized the peak area of MS2 generated fragment ions, an approach developed in our laboratory [11], to quantify relative changes in PPP1R12B phosphorylation after insulin treatment.

## Results

**Figure 1:**
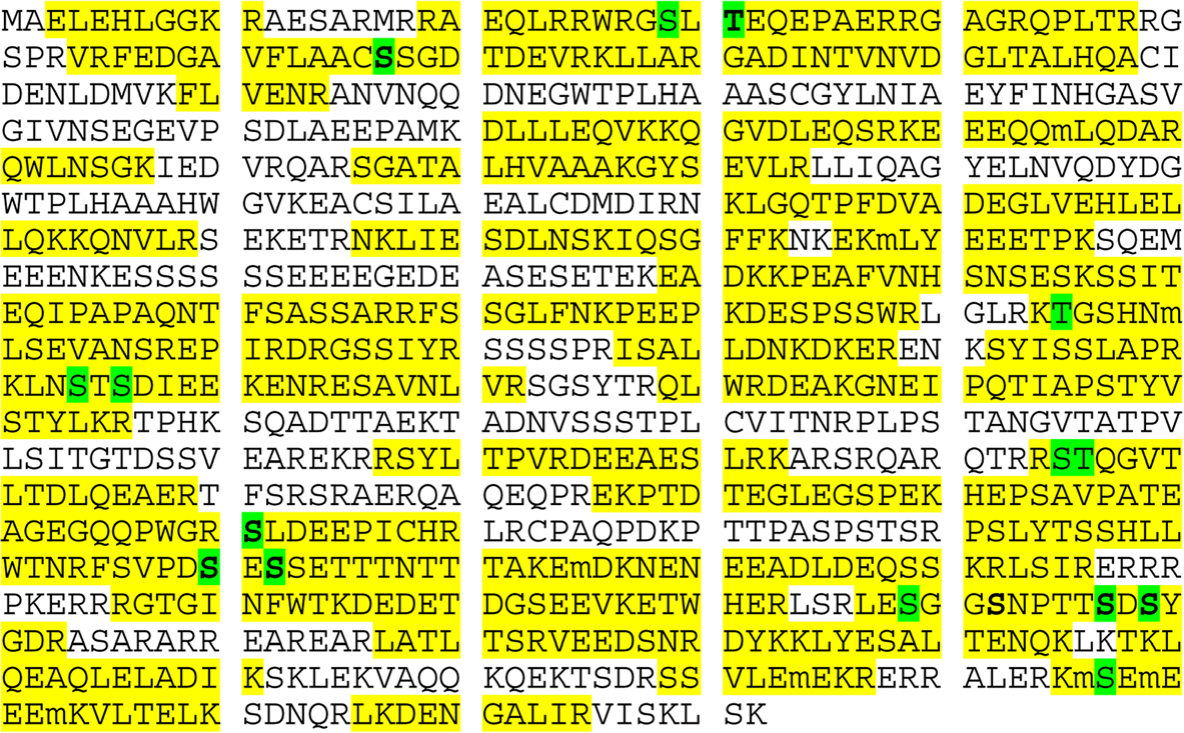
**Combined coverage map of peptides detected in tryptic digests of PPP1R12B expressed in CHO/IR cells using HPLC-ESI-MS/MS analysis.** 63% PPP1R12B sequence coverage was obtained from the 6 basal and 6 insulin samples as well as several preliminary experiments for PPP1R12B peptide discovery. Detected peptides are highlighted in yellow; methionine oxidation sites are lowercase; serine/threonine phosphorylation sites are highlighted in green; and novel phosphorylation sites are in boldface type.

We hypothesized that insulin would regulate phosphorylation of PPP1R12B in Chinese hamster ovary cells overexpressing human insulin receptor (CHO/IR). Therefore we set out to identify PPP1R12B phosphorylation sites and assess how they respond to insulin. To that end, overexpressed FLAG-tagged PPP1R12B was isolated from CHO/IR cells by immunoprecipitation, and then HPLC-ESI-MS/MS was performed, as described in the Methods section. The spectra obtained by HPLC-ESI-MS/MS confirmed the presence of PPP1R12B with 63% sequence coverage (Figure 1). Table 1 lists the PPP1R12B phosphopeptides detected by HPLC-ESI-MS/MS and their respective predominant phosphorylation sites. In all, 14 phosphorylation sites were detected, 7 of which were previously not reported as phosphorylation sites in the four large phosphorylation databases, and thus appear to be novel. These novel, previously unknown phosphorylation sites include Thr31, Ser67, Ser711, Ser760, Ser762, Ser847, and Ser849. Phosphorylation of PPP1R12B at Thr646, observed in kidney cells by Okamoto *et al*. [5], was confirmed in CHO/IR cells; however, based on the tandem mass spectra, the peptide containing phosphorylated Thr646 might also be phosphorylated at Ser645 [12,13]. We confirmed the phosphorylation of PPP1R12B at Ser29 [14], Ser445 [14], Ser504 [14], Ser506 [15], Ser839 [14,16], and Ser947 [14,17]. The MS/MS spectra for the peptides containing phosphorylated Ser645/Thr646 and Ser760 are shown in Additional file 1: Figure S1 and Figure S2. We have posted the Scaffold file on http://cphs-web.cphs.wayne.edu/zyi/PPP1R12B so that readers can access all MS/MS spectra after installation of the Scaffold viewer, which is freely available on http://www.proteomesoftware.com.

**Table 1:** PPP1R12B phosphopeptides and predicted potential kinases

**Start**	**Stop**	**Peptide sequence**	**Phosphorylation site**	Predicted kinases*
28	38	GpSLTEQEPAER	Ser29^†^	CK2α, CK2α’, DMPK, PAK-1, PAK-2, PAK-3, PAK-4, PKAα, PKAβ, p70-S6K
		GSLpTEQEPAER	**Thr31**^**‡**^	CK2α, CK2α’
54	76	VRFEDGAVFLAACpSSGDTDEVRK	**Ser67**	CK2α, CK2α’
445	458	pTGSHNMLSEVANSR	Thr445	DMPK, NEK2
501	511	KLNpSTSDIEEK	Ser504^†^	CK2α, CK2α’
		KLNSTpSDIEEK	Ser506	CK2α, CK2α’
645	659	pSpTQGVTLTDLQEAER	Ser645/Thr646^†^	AIM1, PAK-1, PAK-2, PAK-3, PKAα, PKAβ, DMPK, Pim-2h/ DMPK, Pim-2h, RhoK
711	720	pSLDEEPICHR	**Ser711**	CK2α, CK2α’
755	773	FSVPDpSESSETTTNTTTAK	**Ser760**	CK2α, CK2α’
		FSVPDSEpSSETTTNTTTAK	**Ser762**	CK2α, CK2α’, TLK1
837	853	LEpSGGSNPTTSDSYGDR	Ser839	ActRIIA, ActRIIB, CLK1, CLK2, DMPK, RAGE-1, TGFR-2
		LESGGSNPTTpSDSYGDR	**Ser847**	CK2α, CK2α’
		LESGGSNPTTSDpSYGDR	**Ser849**	ActRIIA, ActRIIB, TGFR-2
945	954	KMpSEMEEEMK	Ser947	CK2α, CK2α’

* Using NetworKIN 2.0 Beta (http://networkin.info/version_2_0/newPrediction.php) as well as literature searches.

^†^ Insulin-stimulated sites.

^‡^ Novel phosphorylation sites are indicated in **boldface type**.

Kinase abbreviations: CK2α, casein kinase II, alpha chain; CK2α’, casein kinase II, alpha' chain; DMPK, myotonic dystrophy protein kinase; PAK-1, p21-activated kinase 1; PAK-2, p21-activated kinase 2; PAK-3, p21-activated kinase 3; PAK-4, p21-activated kinase 4; PKAα, cAMP-dependent protein kinase, alpha-catalytic subunit; PKAβ, cAMP-dependent protein kinase, beta-catalytic subunit; p70-S6K, ribosomal protein S6 kinase 1; NEK2, nimA-related protein kinase 2; AIM-1, Aurora- and Ipl1-like midbody-associated protein 1; Pim-2h, serine/threonine-protein kinase Pim-2; RhoK, Rho kinase, TLK1, tousled-like kinase 1; ACTRIIA, activin receptor type II precursor; ACTRIIB, activin receptor type IIB precursor; CLK1, CDC-like kinase 1; CLK2, CDC-like kinase 2; RAGE-1, renal tumor antigen 1; TGFR-2, TGF-beta receptor type II precursor.

To assess the effect of insulin on PPP1R12B phosphorylation, serum-starved, CHO/IR cells overexpressing FLAG-tagged PPP1R12B were either left untreated or treated with insulin. FLAG-tagged PPP1R12B was immunoprecipitated and resolved by 10% SDS-PAGE. Coomassie blue stain was used to visualize the protein ( Additional file 1: Figure S3), after which the gel area corresponding to PPP1R12B was excised and subjected to trypsin digestion. Relative quantification of phosphorylation by HPLC-ESI-MS/MS was performed as described in the Methods section. Six independent biological replicates (6 control and 6 insulin-treated, total 12 samples) were utilized to increase the confidence of our findings. The control and insulin-stimulated samples that were harvested on the same day, resolved on the same gel, and analyzed by HPLC-ESI-MS/MS during the same period of time were paired to minimize day-to-day variations. Eight nonphosphorylated PPP1R12B peptides were used as endogenous internal standards to measure total PPP1R12B present per sample (Table 2) and their peak area and retention times are listed in Additional file 2: Table S1.

**Table 2:** PPP1R12B internal standard peptides

**Start**	**Stop**	**Peptide sequence**	**Molecular weight (Da)**	Quantified (*m/z*)*
282	303	LGQTPFDVADEGLVEHLELLQK	2451.2766	817.7637 (+3)
				1226.1419 (+2)
327	333	IQSGFFK	826.4458	413.7265 (+2)
				826.4458 (+1)
397	417	SSITEQIPAPAQNTFSASSAR	2163.0677	721.6941 (+3)
				1082.0375 (+2)
492	500	SYISSLAPR	993.5364	497.2718 (+2)
				993.5364 (+1)
515	522	ESAVNLVR	887.4945	444.2509 (+2)
				887.4945 (+1)
741	754	PSLYTSSHLLWTNR	1674.8598	558.9581 (+3)
				837.9336 (+2)
837	853	LESGGSNPTTSDSYGDR	1742.7464	871.8768 (+2)
966	975	LKDENGALIR	1128.6371	564.8222 (+2)
				1128.6371 (+1)

^*^m/z, mass-to-charge ratio.

Combined peak areas for the +1, +2, and +3 (when detected) charge states for each peptide were obtained by integration of the appropriate reconstructed ion chromatograms.

**Figure 2:**
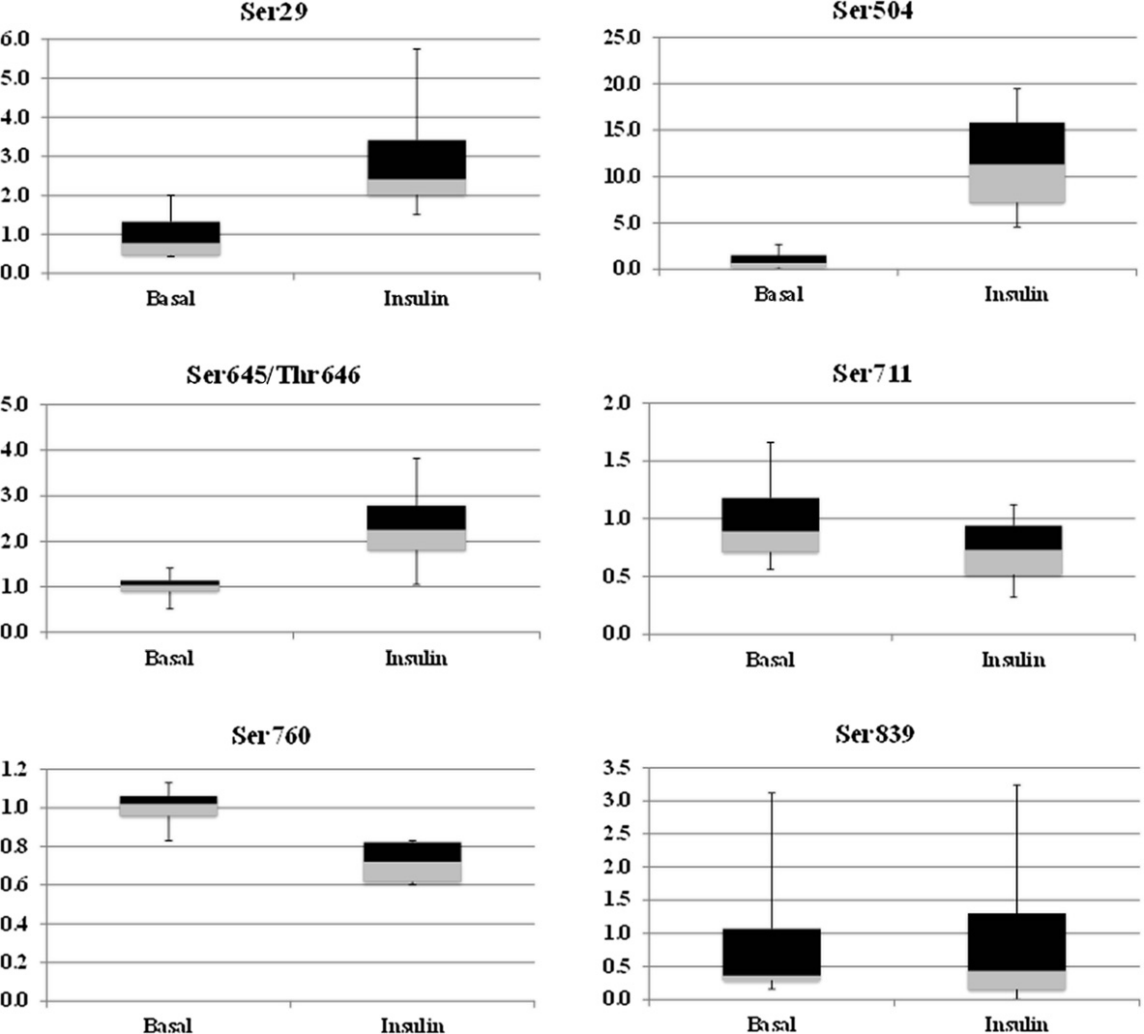
**The effect of insulin on PPP1R12B phosphorylation.** Immunoprecipitated FLAG-tagged PPP1R12B was resolved by 10% SDS-PAGE and stained with Coomassie blue to visualize the protein. The gel area corresponding to PPP1R12B was excised and subjected to trypsin digestion and HPLC-ESI-MS/MS analysis as described in the Methods section. Relative quantification of each phosphopeptide was obtained by comparing normalized peak-area ratios against the average of 8 representative PPP1R12B peptides. Each PPP1R12B phosphorylation site was normalized by the average value of the respective basal sample and then expressed as fold change over control (n = 4). Box and whisker plots of the ratios were utilized to visualize the effect of insulin on PPP1R12B phosphorylation.

Analysis of PPP1R12B phosphorylation revealed that several PPP1R12B phosphopeptides contain multiple phosphorylation sites (Table 1). To quantify the phosphorylated peptides, we generated MS2 fragment ions and used the peak areas of the fragment b and y ions (Table 3), as described by Langlais *et al*. [11]. Among the 14 phosphorylation sites identified, we obtained quantitative information for 6 of them (Figure 2 and Table 4). Please note that even though we performed 6 independent comparisons between basal and insulin treated conditions, 2 of the comparisons had a relatively larger deviation from the other 4 comparisons. Therefore, they were excluded from Figure 2 and Table 4. Nonetheless, biological findings regarding insulin stimulation for 6 comparisons are the same as those for 4 comparisons. Each PPP1R12B phosphorylation site was normalized by the average value of the respective control sample and then expressed as fold change over control ± SEM (n = 4). Phosphorylation of PPP1R12B at Ser711, Ser760, and Ser839 was not significantly affected by insulin (Figure 2 and Table 4). In contrast, significant insulin stimulation was observed for the phosphorylation of PPP1R12B at Ser29, Ser504, and Ser645/Thr646 (Figure 2 and Table 4). Unfortunately, we were unable to discern between the isobaric peptides of aa645-659 that are phosphorylated at either Ser645 or Thr646, as the respective y14 and y15 ions were not readily detectable (which explains why thus were not included in Table 3). We have been forced to group the quantification of these 2 phosphorylation sites together. After correcting for sample loading by dividing the peak area for each phosphopeptide by the corresponding mean peak area of PPP1R12B representative peptides from each sample, these three sites showed an increase in all 4 comparisons after insulin treatment (*P* < 0.05). Phosphorylation increased 3.02 ± 0.94 fold at Ser29, 11.67 ± 3.33 fold at Ser504, and 2.34 ± 0.58 fold at Ser645/Thr646. The increased phosphorylation of PPP1R12B after insulin stimulation has not been previously reported for these sites. We performed a literature search and also utilized NetworKIN 2.0, an online bioinformatics tool, to predict kinases capable of phosphorylating PPP1R12B (Table 1) [18,19]. The potential kinases for the PPP1R12B phosphorylation sites that underwent insulin stimulation (i.e., Ser29, Ser504, and S695/Thr646) included p21-activated kinases (PAKs), casein kinase II (CK2), myotonic dystrophy protein kinase (DMPK), and Rho kinase, all of which have been shown to be activated by insulin [20-22]. Future studies will test the involvement of these kinases in insulin-stimulated phosphorylation of PPP1R12B.

**Table 3:** Unique fragment ions used to quantify phosphorylation sites

**Start**	**Stop**	**Peptide sequence**	**Phosphorylation site**	Product ion (*m/z*)*	**Mass (Da)**
28	38	GpSLTEQEPAER	Ser29	y8 (+1)	959.40
				y9 (+1)	1072.50
501	511	KLNpSTSDIEEK	Ser504	b4 (+1)	523.23
				y7 (+1)	821.39
645	659	pSpTQGVTLTDLQEAER	Ser645/Thr646	b2 (+1)	269.05
				b3 (+1)	397.11
				b4 (+1)	454.13
				b5 (+1)	553.20
				y10 (+1)	1175.59
				y11 (+1)	1274.66
				y12 (+1)	1331.68
				y13 (+1)	1459.74
711	720	pSLDEEPICHR	Ser711	y6 (+1)	754.37
				y7 (+1)	883.41
				y8 (+1)	998.44
755	773	FSVPDpSESSETTTNTTTAK	Ser760^†^	b7 (+1)	842.30
				y12 (+1)	1241.59
				y13 (+1)	1370.63
837	853	LEpSGGSNPTTSDSYGDR	Ser839	b3 (+1)	410.10
				y12 (+1)	1299.50
				y13 (+1)	1356.60
				y14 (+1)	1413.60

^*^m/z, mass-to-charge ratio.

^†^ Since Scaffold PTM analysis indicated that only Ser760 or Ser762 is phosphorylated on this peptide, unique fragment ion selection was based on the comparison between pSer760 and pSer762.

**Table 4:** Effect of insulin on PPP1R12B phosphorylation, n = 4

**Ser29**				
				
Experiment	Normalized peak area ^#^		Ratio ^†^	
	Control	Insulin	Control	Insulin
1	1.10E-05	3.89E-05	0.43	1.51
2	1.20E-05	6.84E-05	0.46	2.65
3	2.85E-05	5.64E-05	1.10	2.18
4	5.18E-05	1.48E-04	2.01	5.75
Average	2.58E-05		1.00	3.02*
SEM			0.37	0.94
**Ser504**				
Experiment	Normalized peak area ^#^		Ratio ^†^	
	Control	Insulin	Control	Insulin
1	2.12E-06	7.66E-05	0.22	8.06
2	2.53E-05	1.85E-04	2.66	19.45
3	1.06E-05	1.39E-04	1.11	14.62
4	0.00E + 00	4.33E-05	0.00	4.56
Average	9.51E-06		1.00	11.67*
SEM			0.60	3.33
**Ser645/T646**				
Experiment	Normalized peak area ^#^		Ratio ^†^	
	Control	Insulin	Control	Insulin
1	1.50E-04	3.00E-04	0.52	1.05
2	2.93E-04	5.89E-04	1.02	2.05
3	3.01E-04	7.03E-04	1.05	2.45
4	4.04E-04	1.10E-03	1.41	3.82
Average	2.87E-04		1.00	2.34*
SEM			0.18	0.58
**Ser711**				
Experiment	Normalized peak area ^#^		Ratio ^†^	
	Basal	Insulin	Basal	Insulin
1	4.15E-05	4.29E-05	0.56	0.58
2	5.58E-05	2.36E-05	0.76	0.32
3	7.55E-05	8.23E-05	1.02	1.12
4	1.22E-04	6.52E-05	1.66	0.88
Average	7.37E-05		1.00	0.73
SEM			0.24	0.17
**Ser760**				
Experiment	Normalized peak area ^#^		Ratio ^†^	
	Basal	Insulin	Basal	Insulin
1	3.43E-05	2.13E-05	1.00	0.62
2	3.59E-05	2.85E-05	1.04	0.83
3	2.84E-05	2.83E-05	0.83	0.82
4	3.89E-05	2.08E-05	1.13	0.60
Average	3.44E-05		1.00	0.72
SEM			0.06	0.06
**Ser839**				
Experiment	Normalized peak area ^#^		Ratio ^†^	
	Basal	Insulin	Basal	Insulin
1	1.36E-05	1.67E-05	0.16	0.20
2	2.74E-05	5.48E-05	0.33	0.66
3	3.23E-05	0.00E + 00	0.39	0.00
4	2.61E-04	2.70E-04	3.12	3.24
Average	8.36E-05		1.00	1.02
SEM			0.71	0.75

^#^ Normalized peak area: obtained by dividing the peak area of a phosphopeptide by the mean value of the peak area of the 8 unphosphorylated representative peptides of PPP1R12B.

^†^ Ratio: obtained by dividing the normalized peak area for a phosphopeptide in each experiment by the average of the normalized peak area under the control condition.

* P < 0.05 compared with control.

## Discussion

It has been shown that phosphorylation of PPP1R12B at Thr646 by Rho kinase reduces the activity of the PPP1R12B-PP1cδ complex against smooth muscle myosin light chain in COS7 kidney cells [5]. Whether Thr646 phosphorylation plays the same inhibitory role in PPP1R12B-PP1cδ complex activity in CHO/IR cells remains to be elucidated. A previous report indicated that insulin might stimulate Rho kinase activity [23]. Thus, it is possible that after insulin stimulation, Rho kinase phosphorylates Thr646 in PPP1R12B in CHO/IR cells and serves as a negative regulator of the PPP1R12B-PP1cδ complex. We also observed the phosphorylation of PPP1R12B at the pThr646 proximal site, Ser645, although these 2 phospho sites were not distinguishable based on the MS/MS spectrum, and whether they behave similarly in the regulation of PPP1R12B is unclear at present. Mutation of Thr646 or Ser645 to alanine is on-going to assess the role of PPP1R12B phosphorylation on PP1c activity and insulin signaling.

Ser29 and Thr31 are in close proximity to the PP1c binding motif (K/R-I/V-X-F/W, residues 53–56 in human PPP1R12B) [5]. In addition, the crystalline structure of the PP1 complex between the chicken PP1c δ isoform (also called β isoform) and amino acids 1–299 of protein phosphatase 1 regulatory subunit 12A (PPP1R12A) (also called MYPT1, an isoform of PPP1R12B) has been resolved [24]. It indicates that residues 1–34, which precede the PP1c binding motif in human PPP1R12A (residues 35–38), also interact with PP1cδ. It has been shown that a short peptide (residues 23–38) of PPP1R12A, which contains the PP1c binding motif but lacks the N terminus, binds to PP1c but has no effect on PP1cδ catalytic activity [25], whereas a peptide containing residues 1–38 of PPP1R12A both interacts with PP1c and increases its phosphatase activity. Hence, it is reasonable to conclude that some structure within residues 1–22 is responsible for the increased catalytic activity. To date, structural information for PPP1R12B is lacking. However, based on the similarity between PPP1R12A and PPP1R12B as well as the insulin-stimulated phosphorylation of Ser29 (pS29), we speculate that pS29 might play a role in regulating PP1cδ activity when it is in a complex with PPP1R12B. Without pS29, PPP1R12B might still bind to PP1cδ through the PP1c binding motif; however, the resulting complex might not have the full phosphatase activity against its substrates. We are in the process of mutating Ser29 to alanine to test the functional consequence of this mutation, such as effect on phosphatase activity.

Ser504 of PPP1R12B exhibited over 11-fold more phosphorylation after insulin treatment. Because it was found, by surface plasmon resonance, that PP1cδ might interact with the PPP1R12A truncation containing residues 304–511 [25], we speculate that phosphorylation at Ser504 might also be involved in the interplay between PPP1R12B and PP1cδ. The increase in phosphorylation of PPP1R12B at Ser504 represents the strongest fold change of any insulin-stimulated serine or threonine phosphorylation site that we have studied to date using this mass spectrometry technique to quantify protein phosphorylation [11,26-28]. The strength of the insulin-stimulated PPP1R12B phosphorylation at Ser504 could indicate that it is a major regulatory mechanism responsible for controlling PPP1R12B function in insulin signaling. Mutation of Ser504 to alanine is on-going to assess the function of this phosphorylation site in regulating PPP1R12B and PP1c activity.

Insulin signaling is crucial to many biological processes, such as glycogen synthesis, glucose transport, mitogenesis, and protein synthesis. The intracellular actions of insulin are mediated by controlled protein phosphorylation and dephosphorylation [29]. Insulin activates the insulin receptor, and the activated insulin receptor then phosphorylates tyrosine residues IRS-1, which allows IRS-1 to recruit phosphatidylinositide 3-kinase and leads to phosphorylation of Akt on threonine/serine residues. Activated Akt phosphorylates its substrate proteins, such as AS160, and promotes GLUT4 translocation to the plasma membrane, leading to enhanced glucose uptake. In addition, activated Akt can increase glycogen synthesis by phosphorylating glycogen synthase kinase 3, and decreasing the phosphorylation of glycogen synthase. Moreover, phosphorylated Akt enhances protein synthesis through serine/threonine phosphorylation of mammalian target of rapamycin and ribosomal protein S6 kinase beta-1 [29]. Furthermore, IRS-1 interacts with growth factor receptor binding protein 2, leading to serine/threonine phosphorylation of a number of signaling proteins in the mitogen-activated protein kinase pathway and subsequent promotion of cell survival and mitogenesis [30]. As discussed above, several of the serine/threonine kinases, such as Akt, mammalian target of rapamycin, ribosomal protein S6 kinase beta-1, glycogen synthase kinase 3, and mitogen-activated protein kinase, have been shown to play a role in insulin signaling. However, a mechanism for serine/threonine phosphatase action in insulin signal transduction is not known. The present study identified PPP1R12B, a regulatory subunit of PP1 (which is a serine/threonine protein phosphatase), as a new insulin-signaling protein with site-specific phosphorylation that is regulated by insulin in CHO/IR cells. The results presented in this study will provide targets for future investigations delineating the role of serine/threonine phosphatases in insulin signaling.

## Conclusions

**Figure 3:**
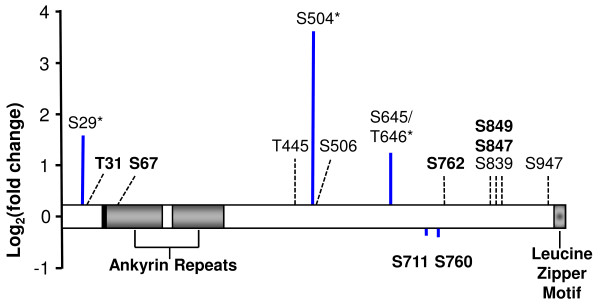
**Summary of PPP1R12B phosphorylation findings.** The black bar preceding the Ankyrin repeats represents the PP1c binding motif, amino acids 53–56. An asterisk (*) indicates sites with a significant increase in phosphorylation after insulin stimulation. Novel sites are indicated in boldface type.

We analyzed the effect of insulin on PPP1R12B phosphorylation using HPLC-ESI-MS/MS and found that insulin stimulated phosphorylation of Ser29, Ser504, and Ser645/Thr646. We also identified 7 previously unreported PPP1R12B phosphorylation sites, namely, Thr31, Ser67, Ser711, Ser760, Ser762, Ser847, and Ser849. Although these novel sites did not respond to insulin in CHO/IR cells, they provide targets for investigating the regulation of PPP1R12B and/or PP1cδ in other cells, such as smooth muscle cells, cardiomyocytes, or COS7 kidney cells. A summary of the PPP1R12B phosphorylation findings is provided in Figure 3. It is noted that overexpression of insulin receptor might lead to artifactual phosphorylation. Nonetheless, these results provide novel targets for future investigation of the regulation of PPP1R12B not only in insulin signaling in cell models, animal models, and in humans, but also in other signaling pathways. Future experiments will confirm the effect of insulin on PPP1R12B phosphorylation in both animal and human muscle, while site-specific mutagenesis will be employed to assess the role of PPP1R12B phosphorylation on PP1c activity and insulin signaling within *in vitro* insulin-signaling models, such as L6 myotubes.

## Methods

### Materials

The sequencing-grade trypsin and anti-FLAG antibody were purchased from Sigma (St. Louis, MO), and the C18 ZipTip from Millipore (Billerica, MA). Chinese hamster ovary cells overexpressing the insulin receptor (CHO/IR) were a gift from Dr. Feng Liu (University of Texas Health Science Center at San Antonio, TX). Establishment of the CHO/IR cell line was described previously [31]. The cDNA encoding full-length wild-type human PPP1R12B was a gift from Dr. Ryuji Okamoto and Dr. Masaaki Ito (Mie University, Tsu, Mie, Japan).

### Cell culture, transfection, immunoprecipitation, and SDS-PAGE

CHO/IR cells were transfected with 5–10 μg of FLAG-tagged PPP1R12B plasmid DNA using Lipofectamine reagent (Invitrogen, Carlsbad, CA), serum starved for 4 h at 37°C, and left untreated or treated with insulin (100 nM) for 15 min at 37°C. The cells were lysed, and cell lysates (1 mg) were diluted in lysis buffer and incubated with 2 μg of anti-FLAG antibody for PPP1R12B purification. The immunoprecipitates were collected with Protein A agarose beads (Sigma, St. Louis, MO). Samples were boiled in sodium dodecyl sulfate-polyacrylamide gel electrophoresis (SDS-PAGE) sample buffer and resolved by 10% 1D-SDS-PAGE. The proteins were then visualized by Coomassie blue staining (Sigma, St. Louis, MO). Please see Additional file 3 for more details.

### In-gel digestion and mass spectrometry

In-gel digestion and mass spectrometry were performed as described previously [11,26,32]. Briefly, the gel portions containing PPP1R12B were excised, destained, dehydrated, dried, and subjected to trypsin digestion overnight. The resulting peptides were desalted and analyzed by on-line HPLC on a linear trap quadrupole-Fourier transform ion cyclotron resonance (LTQ-FTICR). Please see the Additional file 3 for details.

Phosphorylation sites were located using Scaffold PTM (version 1.0.3, Proteome Software, Portland, OR), a program based on the Ascore algorithm [33,34]. Sites with Ascores ≥ 13 (*P* ≤ 0.05) were considered confidently localized [33,34].

Peak areas for each peptide were obtained by integrating the appropriate reconstructed ion chromatograms with 10 ppm error tolerance for precursor-ion masses acquired using FTICR and 0.5 Dalton for the fragment ions acquired using the LTQ mass analyzer. Relative quantification of each phosphopeptide was obtained by comparing normalized peak-area ratios for control and insulin-treated samples [11,26,32].

### Statistical analysis

Statistical significance was assessed by comparing control and insulin-stimulated phosphopeptide peak areas (normalized to PPP1R12B representative peptides as described above) using the paired *t*-test.

## Abbreviations

CHO/IR, Chinese hamster ovary cells overexpressing human insulin receptor; HPLC-ESI-MS, High-performance liquid chromatography-electrospray ionization-mass spectrometry; IRS-1, Insulin receptor substrate-1; LTQ-FTICR, Linear trap quadrupole-Fourier transform ion cyclotron resonance; MS/MS, Tandem mass spectrometry; PP1c, Protein phosphatase 1 catalytic subunit; PPP1R12B, Protein phosphatase 1 regulatory subunit 12B.

## Competing interests

The authors declare that they have no competing interests.

## Authors’ contributions

KP carried out the experiments, analyzed the data, and wrote the manuscript; PL organized and analyzed the data, and wrote and revised the manuscript; XZ wrote and revised the manuscript; AC analyzed the data; MZ carried out the experiments; ZY conceived and designed the experiments, analyzed the data, wrote and revised the manuscript. All authors approved the final version of the article.

## Supplementary Material

Additional file 1 **Figure S1.** Tandem mass spectrum of Ser645/Thr646 (ambiguous) phosphorylation in PPP1R12B tryptic phosphopeptide 645-659, pSpTQGVTLTDLQEAER, as well as the theoretical and experimental m/z values for detected fragment ions. *Loss of H_3_PO_4_ (98 units) from the indicated fragment. ^#^Loss of H_2_O (18 units). The ions corresponding to b4, b5, y10, y11, and y12 are consistent with phosphorylation on Ser645/Thr646. **Figure S2.** Tandem mass spectrum of Ser760 phosphorylation in PPP1R12B tryptic phosphopeptide 755-773, FSVPDpSESSETTTNTTTAK as well as the theoretical and experimental m/z values for detected fragment ions. *Loss of H_3_PO_4_ (98 units) from the indicated fragment. ^#^Loss of H_2_O (18 units). The ions corresponding to b5, b6, y11, y12, and y13 are consistent with phosphorylation on Ser760. **Figure S3.** The representative image of the stained gel from which the bands were excised. Click here for file

Additional file 2 **Table S1.** PPP1R12B representative unphosphorylated peptides peak area and retention time. Click here for file

Additional file 3 Supplemental materials and methods. Click here for file
