# Supplementary material for: Insulin-stimulated phosphorylation of protein phosphatase 1 regulatory subunit 12B revealed by HPLC-ESI-MS/MS
Source: Proteome Sci. 2012 Sep 1;10:52. doi: 10.1186/1477-5956-10-52 (PMC3546068; doi:10.1186/1477-5956-10-52)

**Supplemental Figure 1. Tandem mass spectrum of Ser645/Thr646 (ambiguous) phosphorylation in PPP1R12B tryptic phosphopeptide 645-659, pSpTQGVTLTDLQEAER, as well as the theoretical and experimental m/z values for detected fragment ions.** *Loss of H_3_PO_4_ (98 units) from the indicated fragment. ^#^Loss of H_2_O (18 units). The ions corresponding to b4, b5, y10, y11, and y12 are consistent with phosphorylation on Ser645/Thr646.


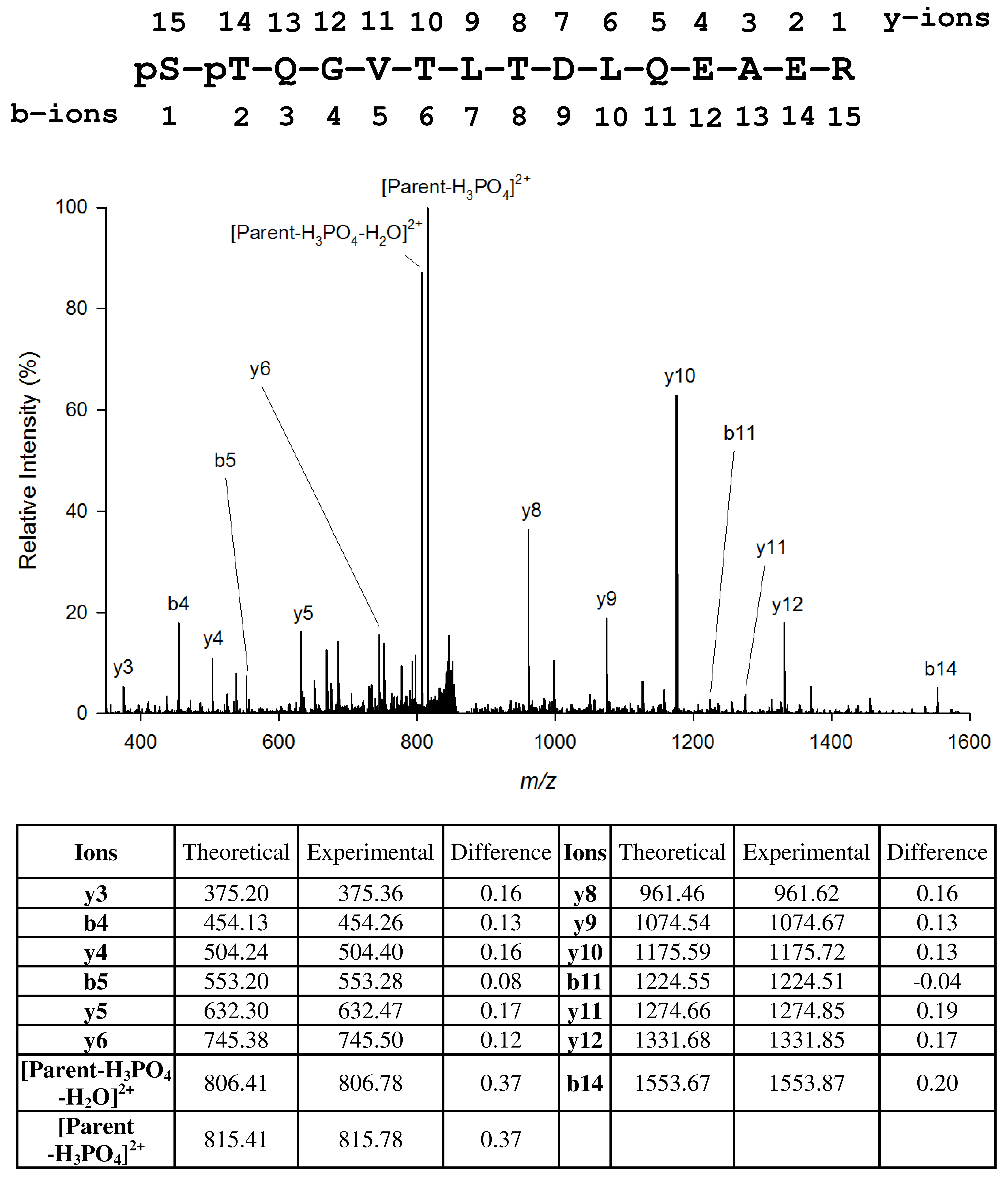


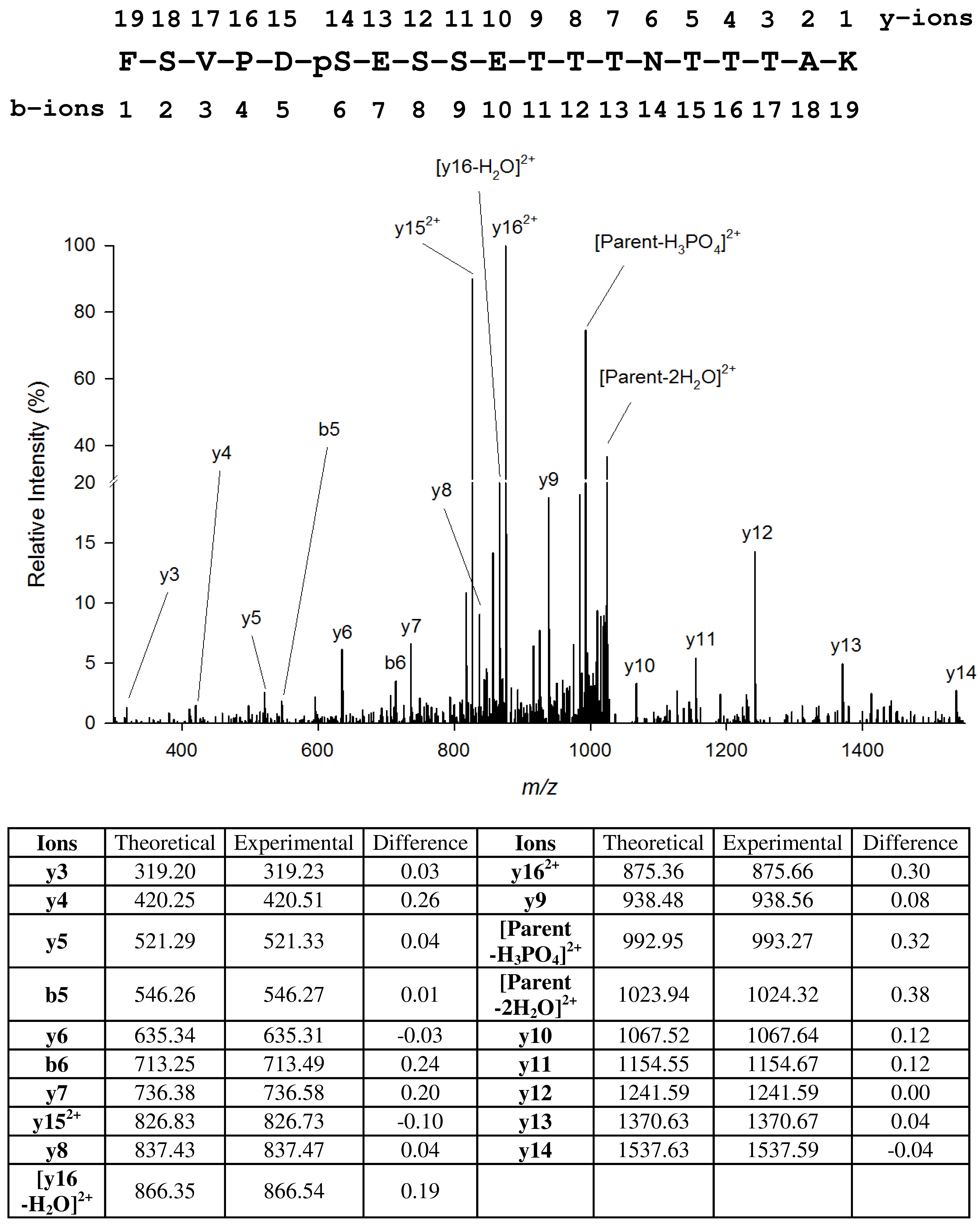
Supplemental Figure 2. Tandem mass spectrum of Ser760 phosphorylation in PPP1R12B tryptic phosphopeptide 755-773, FSVPDpSESSETTTNTTTAK as well as the theoretical and experimental m/z values for detected fragment ions. *Loss of H_3_PO_4_ (98 units) from the indicated fragment. ^#^Loss of H_2_O (18 units). The ions corresponding to b5, b6, y11, y12, and y13 are consistent with phosphorylation on Ser760.

**Supplemental Figure 3.** **The representative image of the stained gel from which the bands were excised.**


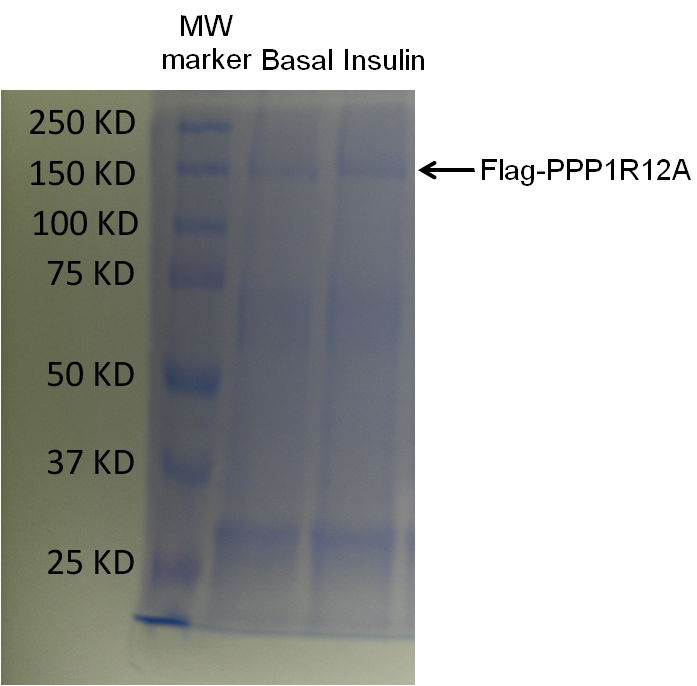

Supplement: Additional file 1 — Figure S1. Tandem mass spectrum of Ser645/Thr646 (ambiguous) phosphorylation in PPP1R12B tryptic phosphopeptide 645-659, pSpTQGVTLTDLQEAER, as well as the theoretical and experimental m/z values for detected fragment ions. *Loss of H3PO4 (98 units) from the indicated fragment. #Loss of H2O (18 units). The ions corresponding to b4, b5, y10, y11, and y12 are consistent with phosphorylation on Ser645/Thr646. Figure S2. Tandem mass spectrum of Ser760 phosphorylation in PPP1R12B tryptic phosphopeptide 755-773, FSVPDpSESSETTTNTTTAK as well as the theoretical and experimental m/z values for detected fragment ions. *Loss of H3PO4 (98 units) from the indicated fragment. #Loss of H2O (18 units). The ions corresponding to b5, b6, y11, y12, and y13 are consistent with phosphorylation on Ser760. Figure S3. The representative image of the stained gel from which the bands were excised. [file 1477-5956-10-52-S1.docx]
