# Supplementary material for: Insulin-stimulated phosphorylation of protein phosphatase 1 regulatory subunit 12B revealed by HPLC-ESI-MS/MS
Source: Proteome Sci. 2012 Sep 1;10:52. doi: 10.1186/1477-5956-10-52 (PMC3546068; doi:10.1186/1477-5956-10-52)
Supplement: Additional file 3 — Supplemental materials and methods. [file 1477-5956-10-52-S3.docx]

**Cell culture, transfection, immunoprecipitation, and SDS-PAGE**

CHO/IR cells were maintained in Ham’s F-12 medium (Invitrogen, Carlsbad, CA) supplemented with 10% fetal bovine serum and 1% penicillin/streptomycin in a humidified atmosphere containing 5% CO_2_ at 37ºC. Cells were subcultured by trypsinization of subconfluent cultures using 0.25% trypsin with EDTA and were seeded at 2.5 x 10^6^ cells per 10-cm dish. Cells were transfected with 5-10 μg of FLAG-tagged PPP1R12B plasmid DNA using Lipofectamine reagent (Invitrogen, Carlsbad, CA) according to the manufacturer’s protocol. Twenty-four hours after transfection, cells were serum starved for 4 h at 37ºC and left untreated or treated with insulin (100 nM) for 15 min at 37ºC. The cells were lysed with 1 mL of lysis buffer (50 mM HEPES [pH 7.6], 150 mM NaCl, 20 mM sodium pyrophosphate, 10 mM NaF, 20 mM beta-glycerophosphate, 1% Triton, 1 mM Na_3_VO_4_, 1 mM phenylmethylsulfonyl fluoride, and 10 µg/ml leupeptin and aprotinin). Bradford Assay (Bio-Rad) was used to estimate protein concentration using bovine serum albumin (Sigma, St Louis, MO) as a standard. Cell lysates (1 mg) were diluted in lysis buffer and incubated with 2 µg of anti-FLAG antibody for PPP1R12B purification. The immunoprecipitates were collected with Protein A agarose beads (Sigma, St. Louis, MO) overnight at 4°C with gentle rotation and were then washed three times with phosphate-buffered saline. Samples were boiled in sodium dodecyl sulfate-polyacrylamide gel electrophoresis (SDS-PAGE) sample buffer and resolved on 10% 1D-SDS-PAGE. The proteins were then visualized by Coomassie blue staining (Sigma, St. Louis, MO).

**In-gel digestion and mass spectrometry**

All assays were performed as described previously [[1-3](#_ENREF_1)].

***In-gel digestion.*** The gel portions containing PPP1R12B were excised, placed in a 0.6-mL polypropylene tube, destained twice with 300 µL of 50% acetonitrile (ACN) in 40 mM NH_4_HCO_3_, and dehydrated with 100% ACN for 15 minutes. After removal of ACN by aspiration, the gel pieces were dried in a vacuum centrifuge at 60°C for 30 minutes. Trypsin (250 ng; Sigma, St. Louis, MO) in 20 µL of 40 mM NH_4_HCO_3_ was added, and the samples were maintained at 4°C for 15 minutes prior to the addition of 50 µL of 40 mM NH_4_HCO_3_ containing three peptides (at 10-fmol/μl each) to serve as internal standards (Bradykinin Fragment 2-9, B1901; β-Sheet Breaker Peptide, S7563; and Anaphylatoxin C3a fragment, A8651; Sigma, St Louis, MO).

The digestion was allowed to proceed at 37ºC overnight and was terminated by adding 10 µL of 5% formic acid (FA). After further incubation at 37°C for 30 minutes and centrifugation for 1 minute, each supernatant was transferred to a clean polypropylene tube. The extraction procedure was repeated using 40 µL of 0.5% FA, and the two extracts were combined. The resulting peptide mixtures were purified by solid-phase extraction (C18 ZipTip) after sample loading in 0.01% heptafluorobutyric acid:5% FA (v/v) and elution with 4 µL of 50% ACN:1% FA (v/v) and 4 µL of 80% ACN:1% FA (v/v), respectively. The eluates were combined and dried by vacuum centrifugation, and 11 µl of 0.1% trifluoroacetic acid with 2% ACN (v/v) were added.

***Mass spectrometry.*** High-performance liquid chromatography–electrospray ionization–mass spectrometry (HPLC-ESI-MS/MS) was performed on a Thermo Finnigan (San Jose, CA) linear trap quadrupole–Fourier transform ion cyclotron resonance (LTQ-FTICR) fitted with a PicoView™ nanospray source (New Objective, Woburn, MA). Online high-performance liquid chromatography (HPLC) was done using a Michrom BioResources Paradigm MS4 micro 2-dimensional HPLC (Alburn, CA) with a PicoFrit™ column (New Objective, Woburn, MA, 75-μm internal diameter, packed with ProteoPep^TM^ II C18 material, 300 Å); mobile phase, linear gradient of 2-27% ACN in 0.1% FA in 65 minutes, with a hold of 5 minutes at 27% ACN, followed by a step to 50% ACN, hold of 5 minutes, and then a step to 80% (flow rate, 400 nl/min).

“Top-10” data-dependent MS/MS analysis was performed to identify PPP1R12B peptides and to obtain their HPLC retention times, as described previously [[3](#_ENREF_3)]. For quantification, we used the following multi-segment strategy: one survey scan, followed by one parent-list collision-induced dissociation (CID) scan and six targeted CID scans. Included in the parent list were the +2 or +3 charge states of the 8 representative PPP1R12B peptides selected from the prominent ions reproducibly observed in the top-10 data-dependent MS/MS analysis. These peptides were used as internal standards for PPP1R12B protein content (see below). They were selected according to the following criteria: (1) they were detected by HPLC-ESI-MS with high intensity among PPP1R12B peptides; (2) no missed cleavage was observed; and (3) there was no methionine in the sequence (to avoid variability due to methionine oxidization) and no N-terminal Gln residues. The +2 ions of the phosphopeptides of interest were placed on the target list. In order to assess the relative quantities of a large number of phosphopeptides in each experiment and still maintain acceptable mass analysis cycle times, the targeted mass-to-charge ratio values were grouped into segments based on their expected HPLC retention times. One main reason for a targeted approach is that it offers the capability to quantify co-eluting isobaric phosphopeptides by using unique fragment ions, as described previously [[1](#_ENREF_1)].

Tandem mass spectra were extracted from Xcalibur ‘RAW’ files and searched against the SwissProt database (taxonomy, human, 20352 entries) using Mascot (Matrix Science, London, UK; version 2.1). Cross-correlation of Mascot search results with X! Tandem was accomplished with Scaffold (Proteome Software, Portland, OR). The search variables that were used were: 10 ppm mass tolerance for precursor-ion masses and 0.5 Da for product-ion masses; digestion with trypsin; a maximum of two missed tryptic cleavages; and variable modifications of oxidation of methionine and phosphorylation of serine, threonine, and tyrosine. Probability assessment of peptide assignments and protein identifications were made by using Scaffold (version Scaffold-01_06_19; Proteome Software, Portland, OR). Only peptides with ≥ 95% probability were considered. Phosphorylation sites were located using Scaffold PTM (version 1.0.3, Proteome Software, Portland, OR), a program based on the Ascore algorithm [[4](#_ENREF_4), [5](#_ENREF_5)]. Sites with Ascores ≥ 13 (*P* ≤ 0.05) were considered confidently localized [[4](#_ENREF_4), [5](#_ENREF_5)]. We will post the Scaffold file on our website so that readers can access all MS/MS spectra after installation of the Scaffold viewer, which is freely available on http://www.proteomesoftware.com.

Peak areas for each peptide were obtained by integrating the appropriate reconstructed ion chromatograms with 10 ppm error tolerance for precursor-ion masses acquired using the FTICR and 0.5D for the fragment ions acquired using the LTQ mass analyzer. Relative quantification of each phosphopeptide was obtained by comparing normalized peak-area ratios for control and insulin-treated samples [[1-3](#_ENREF_1)].

1. Langlais P, Mandarino LJ, Yi Z: **Label-free relative quantification of co-eluting isobaric phosphopeptides of insulin receptor substrate-1 by HPLC-ESI-MS/MS.** *J Am Soc Mass Spectrom* 2010, **21:**1490-1499.

2. Yi Z, Langlais P, De Filippis EA, Luo M, Flynn CR, Schroeder S, Weintraub ST, Mapes R, Mandarino LJ: **Global assessment of regulation of phosphorylation of insulin receptor substrate-1 by insulin in vivo in human muscle.** *Diabetes* 2007, **56:**1508-1516.

3. Yi Z, Luo M, Mandarino LJ, Reyna SM, Carroll CA, Weintraub ST: **Quantification of phosphorylation of insulin receptor substrate-1 by HPLC-ESI-MS/MS.** *J Am Soc Mass Spectrom* 2006, **17:**562-567.

4. Beausoleil SA, Villen J, Gerber SA, Rush J, Gygi SP: **A probability-based approach for high-throughput protein phosphorylation analysis and site localization.** *Nat Biotechnol* 2006, **24:**1285-1292.

5. Zhai B, Villen J, Beausoleil SA, Mintseris J, Gygi SP: **Phosphoproteome analysis of Drosophila melanogaster embryos.** *J Proteome Res* 2008, **7:**1675-1682.
